# G2 checkpoint targeting via Wee1 inhibition radiosensitizes EGFRvIII-positive glioblastoma cells

**DOI:** 10.1186/s13014-023-02210-x

**Published:** 2023-01-29

**Authors:** Meryem H. Cetin, Thorsten Rieckmann, Konstantin Hoffer, Britta Riepen, Sabrina Christiansen, Fruzsina Gatzemeier, Simon Feyerabend, Melanie Schoof, Ulrich Schüller, Cordula Petersen, Martin Mynarek, Kai Rothkamm, Malte Kriegs, Nina Struve

**Affiliations:** 1 Department of Radiobiology & Radiation Oncology, University Medical Center Hamburg-Eppendorf, Martinistr. 52, 20246 Hamburg, Germany; 2 Department of Otolaryngology and Head and Neck Surgery, University Medical Center Hamburg-Eppendorf, Hamburg, Germany; 3 Research Institute Children’s Cancer Center Hamburg, Hamburg, Germany; 4 Department of Pediatric Hematology and Oncology, University Medical Center Hamburg-Eppendorf, Hamburg, Germany; 5 Institute of Neuropathology, University Medical Center Hamburg-Eppendorf, Hamburg, Germany; 6 Mildred-Scheel Cancer Career Center HaTriCs4, University Medical Center Hamburg-Eppendorf, Hamburg, Germany

**Keywords:** Glioblastoma, EGFRvIII, Wee1 inhibition, Adavosertib, X-irradiation, Radiosensitization

## Abstract

**Background:**

The gene of the Epidermal growth factor receptor (*EGFR*) is one of the most frequently altered genes in glioblastoma (GBM), with deletions of exons 2–7 (EGFRvIII) being amongst the most common genomic mutations. EGFRvIII is heterogeneously expressed in GBM. We already showed that EGFRvIII expression has an impact on chemosensitivity, replication stress, and the DNA damage response. Wee1 kinase is a major regulator of the DNA damage induced G2 checkpoint. It is highly expressed in GBM and its overexpression is associated with poor prognosis. Since Wee1 inhibition can lead to radiosensitization of EGFRvIII-negative (EGFRvIII−) GBM cells, we asked, if Wee1 inhibition is sufficient to radiosensitize also EGFRvIII-positive (EGFRvIII+) GBM cells.

**Methods:**

We used the clinically relevant Wee1 inhibitor adavosertib and two pairs of isogenetic GBM cell lines with and without endogenous EGFRvIII expression exhibiting different *TP53* status. Moreover, human GBM samples displaying heterogenous EGFRvIII expression were analyzed. Expression of Wee1 was assessed by Western blot and respectively immunohistochemistry. The impact of Wee1 inhibition in combination with irradiation on cell cycle and cell survival was analyzed by flow cytometry and colony formation assay.

**Results:**

Analysis of GBM cells and patient samples revealed a higher expression of Wee1 in EGFRvIII+ cells compared to their EGFRvIII− counterparts. Downregulation of EGFRvIII expression by siRNA resulted in a strong decrease in Wee1 expression. Wee1 inhibition efficiently abrogated radiation-induced G2-arrest and caused radiosensitization, without obvious differences between EGFRvIII− and EGFRvIII+ GBM cells.

**Conclusion:**

We conclude that the inhibition of Wee1 is an effective targeting approach for the radiosensitization of both EGFRvIII− and EGFRvIII+ GBM cells and may therefore represent a promising new therapeutic option to increase response to radiotherapy.

**Supplementary Information:**

The online version contains supplementary material available at 10.1186/s13014-023-02210-x.

## Background

Glioblastoma multiforme (GBM; CNS grade 4) is the most common malignant brain tumor in adult patients, with an estimated 5-year survival rate of less than 10% [1]. Since 2005, the current standard of care is an intensive multimodal treatment including neurosurgical resection, radiotherapy (RT) and concomitant and adjuvant chemotherapy (CT) with temozolomide (TMZ) [1, 2]. Despite recent advances in understanding the underlying molecular mechanisms of treatment resistance and response, there has been little improvement in clinical outcome. Hence, there is an urgent need to develop new strategies to enhance the therapeutic effects of radio- and chemotherapy in GBM.

GBMs are generally characterized by inter- and intratumoral heterogeneity with genomic rearrangements and a variety of mutations [3–5]. The most frequent alteration is the amplification of the gene encoding the epidermal growth factor receptor (EGFR), leading to overexpression of EGFR. This gene amplification, which is present in approximately 50% of GBMs, is often associated with the expression of the deletion variant EGFRvIII. This variant lacks the exons 2–7, leading to a ligand-independent and constitutively activated receptor [6, 7]. Recently, we demonstrated that EGFRvIII expression is associated with increased chemosensitivity of GBM cells and tumors. We also showed that EGFRvIII expression is associated with replication stress, R-loop formation and an accumulation of EGFRvIII+ cells in the S/G2 phase of the cell cycle [8]. One major regulator of the DNA damage induced G2-checkpoint is the Wee1 kinase, the overexpression is associated with poor prognosis in GBM [9]. Wee1 phosphorylates the cyclin dependent kinase 1 (CDK1) at amino acid tyrosine 15, an inhibitory phosphorylation, leading to CDK1 inhibition and with that to a block in G2-M transition. Furthermore, Wee1 also phosphorylates CDK2 on tyrosine 15, which leads to decreased CDK2 kinase activity, thereby delaying G1/S phase transition. Therefore, Wee1 inhibition also causes increased replication stress through the upregulation of CDK2 activity and with this excess, unscheduled origin firing and degradation of nascent DNA [10, 11].

Upon X-irradiation, cells induce a protective cell cycle arrest in G1- or G2 phase. While *TP53* proficient normal tissue cells can arrest in G1, the radiation-induced G2-arrest is the only way *TP53* deficient cancer cells can effectively halt cell cycle progression in order to repair DNA double-strand breaks (DSBs) before the critical passage through mitosis [12]. Wee1 inhibition has been shown to radiosensitize cells of different cancer entities, such as head and neck squamous cell carcinoma, esophageal cancer and GBM cells [9, 13, 14]. In GBM, Wee1- inhibition by PD0166285 has shown radiosensitizing effects in EGFRvIII− GBM cells displaying different *TP53* status due to the abrogation of the irradiation induced G2-arrest [9].

Due to its effects on replication stress and cell cycle progression, it is tempting to speculate, that Wee1-inhibition may have pronounced anti-proliferative and radiosensitizing effects in EGFRvIII+ cells, which display higher levels of endogenous replication stress and DNA damage. To test this hypothesis, we explored the effect of the clinically relevant Wee1 inhibitor adavosertib in an established isogenic GBM cell line model system with and without endogenous EGFRvIII expression demonstrating different *TP53* status alone and in combination with x-irradiation.

## Materials and methods

### Inhibitor

Wee1 inhibition was performed using adavosertib (Selleckchem, Houston, TX, USA).

### Cell culture

The human isogenetic EGFRvIII− and EGFRvIII+ GBM sub cell lines DKMGvIII−/+ (*TP53* wildtype) and BS153vIII−/+ (*TP53* mutated) were generated, authenticated and cultivated as described previously [8, 15].

### GBM patient samples

Human tumor material was used in accordance with all local and national ethics guidelines.

### Irradiation

The irradiation of all cells was performed at room temperature with 200 kV X-rays (Gulmay RS225, Gulmay Medical Ltd., 15 mA, 0.8 mm Be + 0.5 mm Cu filtering).

### Cell survival

The ability for self-renewal (clonogenicity) was analyzed by the colony-forming assay as described previously [16]. In brief, 250 cells of DKMGvIII− and DKMGvIII+ cells, 350 cells of BS153vIII− cells and 400 cells of BS153vIII+ cells were seeded per well into a 6-well-plate, 24 h prior the treatment. Cells were treated with adavosertib for 2 h before irradiation. The medium was replaced 24 h after treatment with adavosertib. Cells were further incubated with AmnioMax C-100 Basal Medium (Life Technologies) containing 10% FCS and C-100 supplement (Life Technologies) for optimized colony formation. Cells were grown until the colonies of all treatment arms had reached equal colony size. The number of colonies containing more than 50 cells was assessed.

### Cell proliferation

For cell proliferation analysis, cells were seeded into T25 cell culture flasks and were allowed to grow for 72 h prior to treatment with different adavosertib concentrations. After further incubation for 72 h the cells were fixated and counted with a Beckmann-Coulter (Brea, CA, USA).

### Cell cycle analysis

For cell cycle analysis, cells were seeded into T25 cell culture flasks and were allowed to grow for 24 h prior treatment with different adavosertib and irradiation. Cells were treated with adavosertib and—where indicated—irradiated two hours later. The inhibitor was removed by medium change after 24 h treatment. Cells were harvested, fixed with 70% ethanol and stored at − 20 °C. Propidium iodide staining was performed as described previously. Flow cytometric analysis was performed using a MACSQuant10 with MACSQuantify Software (Miltenyi Biotec, Bergisch Gladbach, Germany). The proportion of cells in the respective cell cycle phases was calculated using ModFit LT™ software (Verity Software House, Topsham, ME, USA).

### Western blot

All proteins were detected by Western blot analysis according to standard protocols. For the detection and quantification, the *Odyssey® CLx Infrared Imaging System* (LI-COR Biosciences) was used. Primary antibodies that were used: EGFR (1:1000, rabbit, Cell Signalling, #2232), β-Actin (1:40000, mouse, Sigma-Aldrich, #A-2228), CDK1 (1:1000, mouse, BD Biosciences, #610037), pCDK1 (1:1000, rabbit, Cell Signalling, #9111), CDK2 (1:1000, mouse, BD Biosciences, #610146), pCDK2 (1:1000, rabbit, GeneTex, #GTX132802), Wee1 (1:1000, rabbit, Cell Signalling, #13084). All primary antibodies were either diluted in 5% bovine serum albumine (BSA) in PBS supplement with 0.2% Tween or with the Intercept Blocking Buffer by LI-COR. The secondary anti-mouse and anti-rabbit antibodies were also purchased by Li-COR Biosciences.

### Cell transfection with EGFRvIII siRNA

Knockdown of EGFRvIII, was performed using HiPerFect (Qiagen, #301705) according to the manufacturer´s instructions. The following siRNA, which covers the novel fusion site in EGFRvIII, was used: EGFRvIII from Eurofins Scientific; [5´-CUGGAGGAAAAGAAAGGUAAU-3´]. On-Target plus Cyclophilin B control pool as control siRNA (Dharmacon, #SO-2436533G). The medium was changed 5 h after transfection.

### Immunohistochemistry

Paraffin-embedded GBM specimens were dewaxed using standard histologic procedure. EGFRvIII (1:250, mouse, Absolute antibody, #Ab00184-1.4) and Wee1 (1:350, rabbit, Cell Signalling, #13084) staining was performed on a Ventana System using standard protocols. Nuclei were counterstained with hematoxylin. For scanning the samples after staining an AxioScan (Zeiss, Axioscan 7 System) was used.

### Immunofluorescence staining

Immunofluorescence staining of EGFRvIII was performed as described previously [15]. For detection of EGFRvIII (L8A4) (1:1000, mouse, #Ab00184-1.1) was used.

### Statistical analysis

Except for immunohistochemistry and unless otherwise indicated, all experiments were repeated at least three times. The data is presented as mean values (SEM). For analyzing and graphing the data Prism software was used. (GraphPad Prism 8, GraphPad Software Inc.). *P*- values were calculated using Student’s *t*-test (*p < 0.05; **p < 0.01).

## Results

**Fig. 1:**
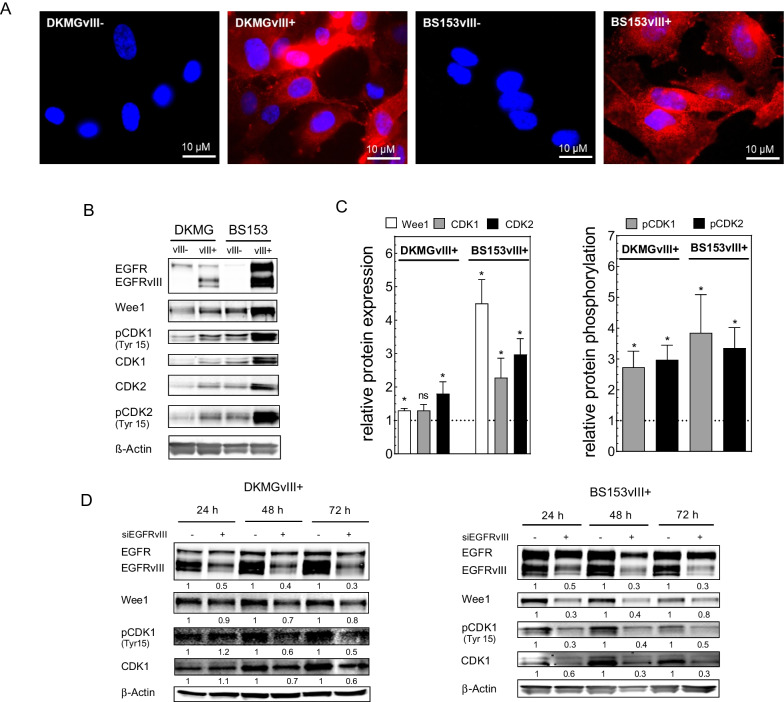
Wee1 expression in EGFRvIII− and EGFRvIII+ GBM cells. **A** EGFRvIII-specific immunofluorescence staining of DKMGvIII− /+ and BS153vIII− /+ cells. **B** Expression respectively phosphorylation of Wee1, CDK1 and CDK2 in DKMGvIII− /+ and BS153vIII− /+ cells. For Western blot analysis, samples were normalized to cell number. β-Actin served as loading control. **C** For quantification of protein expression and phosphorylation, the relative expression/phosphorylation values of EGFRvIII+ cells were normalized to the relative values of EGFRvIII− cells (n = 4; mean with S.E.M; *p*-values are obtained by Mann Whitney test, *p < 0.05). **D** Impact of siRNA-mediated EGFRvIII knockdown in DKMGvIII+ and BS153vIII+ cells on Wee1 and CDK expression and CDK1 phosphorylation after 24 h, 48 h and 72 h. An siRNA against cyclophilin B served as a control

To assess the radiosensitization of GBM cells in dependence of the EGFRvIII status, we used pairs of isogenic GBM sub-cell lines with and without endogenous EGFRvIII expression. These sub-cell lines were isolated from the parental DKMG and BS153 cell lines, both of which display heterogeneous expression of EGFR and EGFRvIII as described previously [8]. As demonstrated in Fig. 1, both EGFRvIII+ sub-strains (DKMGvIII+ and BS153vIII +) were tested positive for EGFRvIII by immunofluorescence (IF) staining (Fig. 1A) and Western Blot analysis (Fig. 1B), whereas their EGFRvIII− counterparts (DKMGvIII− and BS153vIII−) proofed EGFRvIII negative (Fig. 1A, B).

### Wee1 expression in EGFRvIII− and EGFRvIII+ GBM cells

We next analyzed the expression and phosphorylation of Wee1 and its main targets CDK1 and CDK2 in EGFRvIII− and EGFRvIII+ DKMG and BS153 cells by Western blot three days after seeding (Fig. 1B). The quantification revealed a significantly stronger Wee1 expression in both EGFRvIII+ sublines, this effect was especially pronounced in the *TP53* mutated BS153vIII+ cell line. Furthermore, we also observed elevated expression and inhibitory Tyr15 phosphorylation of CDK1 and CDK2 in EGFRvIII+ cells, the latter indicating increased Wee1 activity (Fig. 1C). To assess whether EGFRvIII is the cause for enhanced Wee1 and CDK1 expression as well as Wee1 activity, we transfected the EGFRvIII+ substraines with an EGFRvIII siRNA. Western blot analysis indeed revealed a clear reduction of Wee1 and CDK1 expression and Tyr15 phosphorylated CDK1 (Fig. 1D). It has to be noted, that, despite the use of an EGFRvIII-specific siRNA sequence, at the later time points EGFR wildtype was also downregulated but to a clearly lower extent than EGFRvIII. Overall, these data strongly suggest, that EGFRvIII impacts on Wee1 expression and the phosphorylation of target proteins.

### Wee1 expression in EGFRvIII+ GBM tissue

**Fig. 2:**
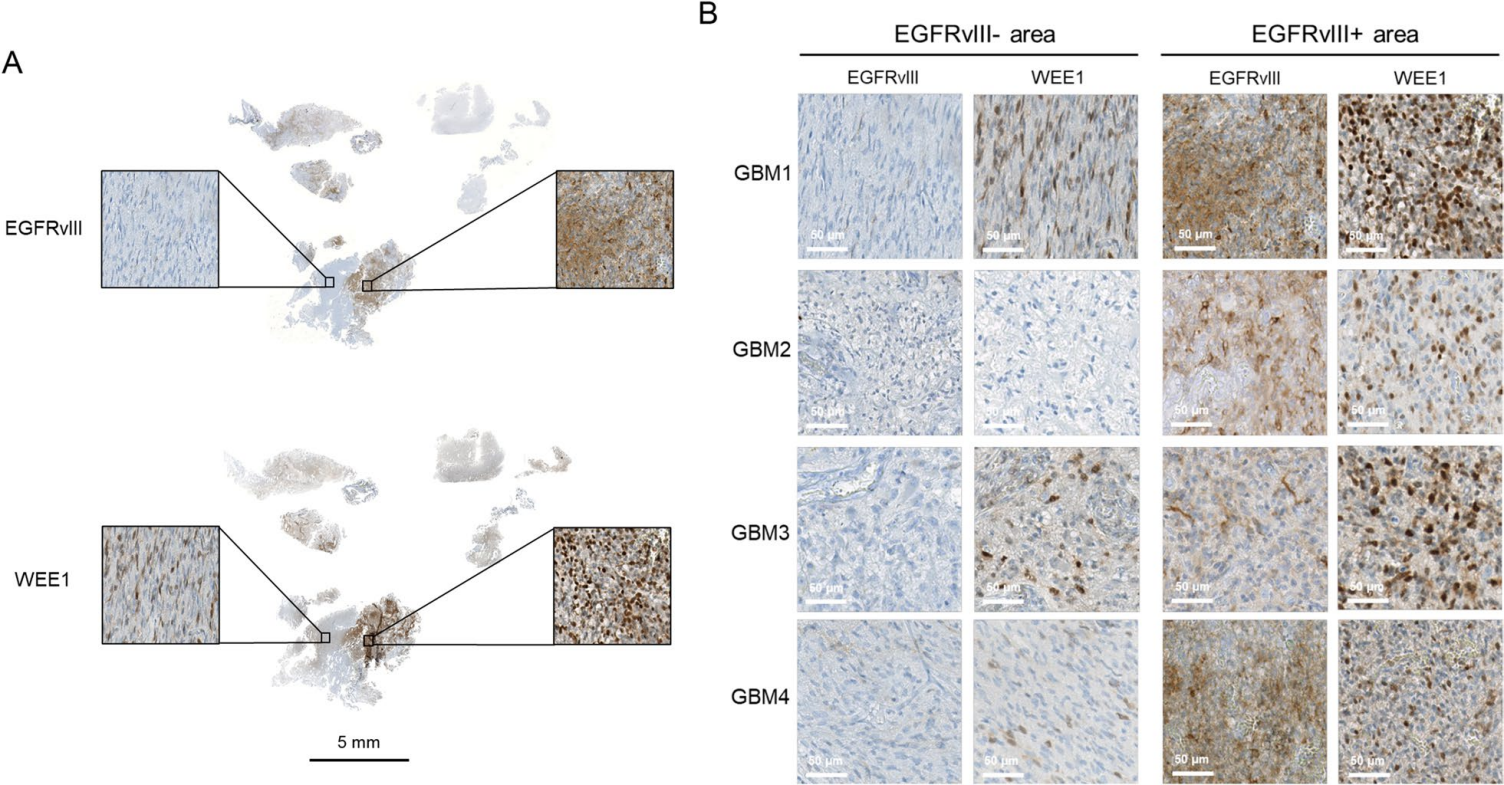
Wee1 expression in EGFRvIII+ GBM patient samples. **A** Immunohistochemical detection of EGFRvIII and Wee1 expression in one representative GBM patient sample (GBM1) displaying heterogeneous EGFRvIII expression. Scale bars represent 50 µm and 5 mm (overview in the middle). **B** Immunohistochemical detection of EGFRvIII and Wee1 expression in four GBM patient samples displaying typical heterogeneous EGFRvIII expression. Scale bars represent 50 µm

To validate the elevated Wee1 and CDK1 expression of EGFRvIII+ cells in human GBM, we analyzed primary patient tissue, using GBM samples displaying typical heterogeneous EGFRvIII expression. Immunohistochemical analysis clearly show increased Wee1 expression in the EGFRvIII+ areas compared to EGFRvIII− areas of the same specimen (Fig. 2A, B; Additional file 1: Fig. 1), demonstrating that our in vitro findings translate into the in situ situation in human GBM tumors.

### Effect of Wee1 inhibition on cell cycle, proliferation and cell survival

To assess the impact of sole Wee1 inhibition on cell cycle, proliferation and survival of DKMGvIII−/+ and BS153vIII−/+ cells, all sub-strains were treated with increasing concentrations of adavosertib up to 1 µM. The treatment had only minor effects on the cell cycle distribution of DKMGvIII−/+ cells showing a small increase in G1 phase cells likely because of a *TP53* dependent arrest in G1 and therefore failure to replenish the S phase. In contrast, at higher concentrations *TP53* mutated BS153vIII−/+ cells showed a decrease in G1 phase cells and an increase in S/G2 phase cells. The BS153vIII− cells showed a stronger and significantly elevated amount of S- and G2 phase cells than the BS153vIII+ cells, probably indicating stronger replication stress (see Additional file 1: Fig. S2). A reason for the different response and cell cycle effects could be the higher expression of Wee1 in BS153vIII+ cells, which could lead to a less efficient inhibition of Wee1. In this regard we observed no pronounced difference in the relative pCDK1 levels in BS153vIII−/+ neither after 6 h nor after 24 h adavosertib treatment. After 24 h the BS153vIII+ cells showed even a stronger relative decrease in pCDK1 levels after Wee1 inhibition, but the absolute levels of pCDK1 were still higher in the BS153vIII+ cells compared to BS153vIII− after Wee1 inhibition, which might affect responsiveness towards AZD treatment (see Additional file 1: Fig. S3). Moreover, the BS153vIII+ cells suffer from endogenous replication stress, which might render the cells less sensitive to exogenous induced replication stress by adavosertib treatment, since these cells already display an activated replication stress response [8].

**Fig. 3:**
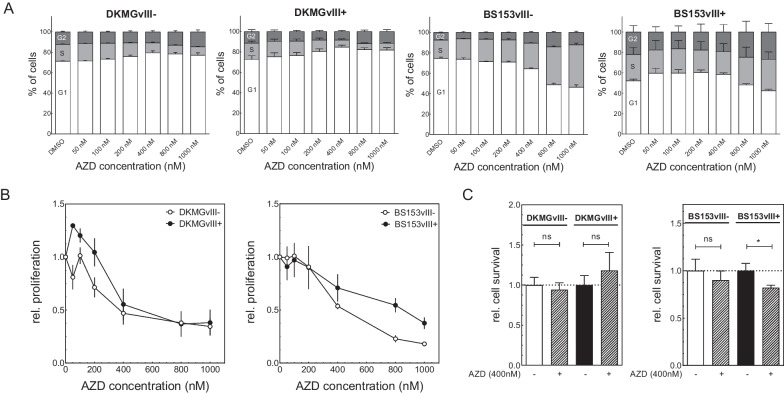
Effect of Wee1 inhibition on proliferation, cell survival and cell cycle of EGFRvIII− /+ cells. **A** Cell cycle distribution was assessed 24 h after adavosertib treatment **B** Effect of increasing adavosertib concentrations on cell proliferation. Treatment with adavosertib was performed for 72 h. **C** Cell survival as measured by colony formation after 400 nm adavosertib treatment. Cells were treated for 24 h with adavosertib (n = 4; mean with S.E.M; *p*-values are obtained by using two-tailed Student´s t-test, *p < 0.05)

For all sub-strains Wee1 targeting inhibited cell proliferation in a dose dependent manner with an IC50 of approximately 400 nM. Although we have recently shown that EGFRvIII+ GBM cells exhibit increased endogenous replication stress, Wee1 inhibition did not lead to a stronger decrease in cell proliferation of EGFRvIII+ cells compared to EGFRvIII− cells. BS153vIII− cells showed, even at higher adavosertib concentrations, enhanced inhibition of proliferation compared to BS153vIII+ cells, whereas DKMGvIII− cells displayed at low adavosertib concentrations a moderately enhanced proliferation (Fig. 3B).

Cytotoxicity of Wee1 inhibition for 24 h was determined using colony formation assays at a concentration of 400 nM adavosertib. For all sub-cell lines except DKMGvIII+ we observed a reduced cell survival after adavosertib treatment. For BS153vIII+ cells this reduction was significant and more pronounced compared to their EGFRvIII− counterpart (BS153vIII−/+ : _DMSO_ 1.0 vs. _AZD_ 0.9/_DMSO_ 1.0 vs. _AZD_ 0.8), suggesting that direct cytotoxicity by Wee1 inhibition might be most efficient in cells displaying high levels of endogenous replication stress [15] (Fig. 3C).

### Effect of Wee1 inhibition on cellular radiosensitivity of EGFRvIII−/+ cells

**Fig. 4:**
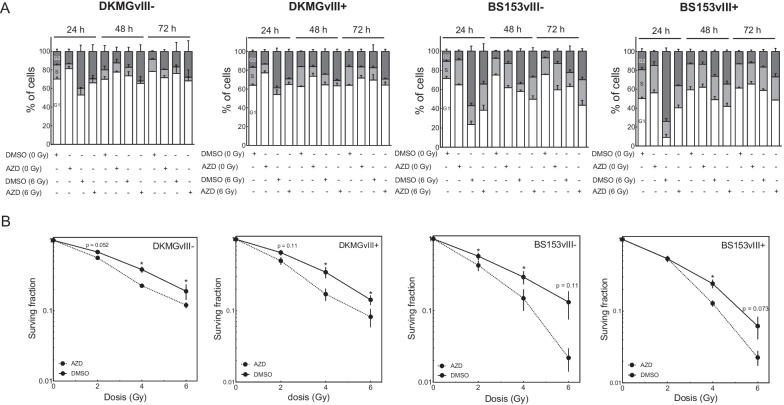
Effect of combined Wee1 inhibition and irradiation on cell survival and cell cycle of EGFRvIII− /+ cells. **A** DKMGvIII− /+ and BS153vIII− /+ cells were treated with 400 nm adavosertib 2 h prior to irradiation with 6 Gy. Cell cycle assessment was performed 24, 48 and 72 h after treatment. **B** DKMGvIII− /+ and BS153vIII− /+ cells were treated with 400 nm adavosertib 2 h prior to irradiation with 0, 2, 4 and 6 Gy. Media exchange was performed 24 h after adavosertib treatment. Cell survival was analyzed by colony formation assay. Absolute colony numbers were normalized to the unirradiated controls (n = 3–5; mean with S.E.M; *p*-values are obtained by using two-tailed Student´s t-test, *p < 0.05)

So far, Wee1 inhibition showed only minor effects on cell cycle distribution and cell survival. However, CDK1/2 inhibition especially occurs after DNA damage, dependent on phosphorylation through Wee1 in concert with CHK1, which, upon activation through DNA damage, inhibits CDK1/2 dephosphorylation through CDC25 proteins. CDK1/2 inhibition then leads to reduced replication rates and especially a transient G2 phase arrest until most of the DNA damage is repaired. In this regard, we combined Wee1 inhibition with x-irradiation for radiosensitizing approaches. As a control we first irradiated DKMGvIII− and DKMGvIII+ cells with 6 Gy and assessed CDK1 phosphorylation up to 48 h. As expected, irradiation induced CDK1 phosphorylation in both cell lines, indicating functional cell cycle checkpoint response (Additional file 1: Fig. 4). For the radiosensitizing approach, cells were treated with adavosertib 2 h prior to irradiation before the media was exchanged after 24 h. First, we analyzed the effect of irradiation with 6 Gy on cell cycle distribution up to 72 h after treatment. Irradiation led to a clear increase in G2 phase cells, which was detectable after 24 h and especially pronounced for *TP53* mutated BS153vIII−/+ cells as compared to *TP53* wildtype DKMGvIII−/+ cells (Fig. 4A). G2-accumulation had declined at 48 h after irradiation, with the DKMGvIII−/+ cells showing nearly a normalization of the cell cycle distribution, whereas BS153vIII−/+ showed a further reduction at 72 h after irradiation. As shown before, treatment with 400 nM adavosertib had little effect in its own but it reduced the irradiation-induced G2 arrest in all cell lines with the most prominent effect observed in BS153vIII+ cells. Here results show a twofold reduction in the amount of G2 cells from 73.8% to 36.2% 24 h after treatment, indicating an inappropriate passage through mitosis in a fraction of cells despite DNA damage levels that would normally still hold cell cycle progression. We observed, a reduction in cell survival for all sub cell lines, irrespective of EGFRvIII status after combination of adavosertib and irradiation demonstrating radiosensitization by Wee1 inhibition (Fig. 4B; DKMGvIII−/+ : SF4Gy_DMSO_ = 0.19 vs. SF4Gy_AZD_ = 0.12/SF4Gy_DMSO_ = 0.35 vs. SF4Gy_AZD_ = 0.17; BS153vIII−/+ : SF4Gy_DMSO_ = 0.29 vs. SF4Gy_AZD_ = 0.14/SF4Gy_DMSO_ = 0.24 vs. SF4Gy_AZD_ = 0.12).

In conclusion, we observed no obvious difference in the extend of radiosensitization between the EGFRvIII− and EGFRvIII+ cell lines, therefore demonstrating that Wee1 inhibition can effectively radiosensitize both EGFRvIII− and EGFRvIII+ GBM cells and could therefore be an efficient new treatment option also for tumors displaying heterogeneous EGFRvIII expression.

## Discussion

Intratumoral EGFRvIII expression in GBM is highly heterogenous. For efficient radiosensitizing approaches it is therefore important to estimate, whether cellular radiosensitivity of both, EGFRvIII− and EGFRvIII+ GBM cells can be increased by specific molecular targeting. The aim of this study was therefore to investigate whether isogenic EGFRvIII−/+ cell lines can be radiosensitized by the clinically relevant Wee1 inhibitor adavosertib. Here, we show that Wee1 inhibition can effectively radiosensitize both, EGFRvIII− and EGFRvIII+ GBM cells (Fig. 4B).

Wee1 inhibition is a promising approach to sensitize various tumor entities towards irradiation since it has been shown to be effective in different cancer cell types such as hepatocellular, head and neck and esophageal cancer [9, 13, 14]. Cuneo et al*.* showed that Wee1 inhibition radiosensitizes hepatocellular carcinoma cell lines regardless of *TP53* mutational status through the induction of replication stress via the overconsumption of nucleotides [17]. Regarding replication stress in GBM, we recently showed, that EGFRvIII expression increases replication stress and the expression and phosphorylation of DNA damage response factors, such as ATR and its downstream effector CHK1. CHK1 phosphorylates and degrades CDC25 phosphatases to prevent activation of CDK1/2 and protect from cell cycle progression before damage is repaired. Therefore, it is reasonable to hypothesize that the altered levels of pCDK1/2 in DKMGvIII+ and BS153vIII+ cells can at least partly be explained by altered CHK1 activity in EGFRvIII+ cells [8]. Overall, we observed a nearly twofold increase in BS153vIII+ cells in the S/G2 phase compared to BS153vIII− cells, which may contribute to the enhanced CDK1/CDK2 and pCDK1/pCDK2 levels. However, to what extent the expression and phosphorylation levels of CDK1 and CDK2 are impacted by the cell cycle in glioblastoma cells is unknown and especially for pCDK1/pCDK2 we observed a higher increase of 3.8 and 3.2-fold in BS153vIII+ cells, which can hardly be explained by the increase in S/G2 phase cells alone (Figs. 1B, C, 3A). Likewise, in DKMGvIII+ cells we observed a 2.8 and threefold increase, while the cell cycle distribution of unperturbed EGFRvIII− respectively EGFRvIII+ cells is basically identical (Fig. 3A).On a functional level, it remains difficult to precisely assess to what extent these changes in expression and activity are caused through EGFRvIII-specific mechanisms or rather through an overall enhanced level of EGFR signaling. Both EGFRvIII+ substrains also express wt-EGFR and knockdown experiments are often specific to a fair extent but rarely completely. Similarly to the situation in our substrains, EGFRvIII expression is basically always associated with EGFR gene amplification in GBM and both variants also interact with one another [18]. It is therefore likely that individual tumors may have different contributions of both EGFR variants, depending on their individual expression and activity patterns.

BS153vIII+ cells, which carry a *TP53* mutation and display an explicitly high expression of EGFRvIII, demonstrated higher levels of replication stress as compared to DKMGvIII+ cells [8]. The elevated expression of Wee1 in both EGFRvIII+ sub-strains demonstrated here, slightly in DKMG and prominently in BS153, may at least in part be a cellular response towards the different levels of EGFRvIII-dependent endogenous replication stress. It is therefore imaginable that due to the already high basal stress level and possibly an enhanced dependence on compensatory Wee1 expression, EGFRvIII+ cells might be more sensitive towards the combined treatment of Wee1 inhibition and irradiation as compared to their EGFRvIII− counterparts. In contrast, Carruthers et al*.* have reported radioresistance in GBM stem cells due to an increased level of endogenous stress and compensatory mechanisms that help the cells to cope with radiation-induced DNA damage [19]. Whether similar compensatory effects could be active in EGFRvIII+ GBM cells and confer resistance to Wee1 inhibition mediated radiosensitization was unknown. Our data now indicate that the extent of radiosensitization did not differ substantially between EGFRvIII− and EGFRvIII+ cells and therefore that neither hypersensitivity nor resistance of EGFRvIII+ tumor fractions can be expected (Fig. 4).

In our study we used inhibitor concentrations, which had been demonstrated to be achievable in the brain respectively tumor and therefore are of therapeutic relevance. In this regard Sanai et al*.* could show in a phase 0 trial, in which patients with recurrent GBM were treated with adavosertib prior to a planned re-operation, that adavosertib reaches therapeutic concentrations within the contrast-enhancing component of the tumor [20]. Notably, these findings differed from preclinical studies using orthotopic GBM xenograft mouse models, which had formerly reported very limited distribution of adavosertib in mouse normal brain and brain tumor tissues. These discrepancies might be based on the fact that recurrent GBM patients in general are heavily pretreated with temozolomide and radiotherapy, which can clearly effect the blood brain barrier integrity. Beyond effective tumor penetration, Sanai et al. could further show that Wee1 inhibition can lead to premature entry into mitosis and ultimately to mitotic catastrophe. Evidence for these adavosertib-dependent effects were observed in patient-derived specimens of recurrent GBM patients who received a single dose of AZD1775 prior to tumor resection. Specifically, at the 8-h posttreatment interval, DNA damage, cell-cycling and apoptosis were increased. Altogether, these data clearly supported further clinical development of adavosertib for treating recurrent glioblastoma [20]. At the moment, a phase I study of adavosertib in combination with radiotherapy and temozolomide in patients with newly diagnosed GBM or recurrent GBM is still ongoing (NCT01849146), emphasizing the importance of radiobiology-related research in this field.

In our in vitro study we observed no differences in the extent of radiosensitization between EGFRvIII− and EGFRvIII+ cells. The degree of radiosensitization also did not correspond to the extent of damage-induced G2-arrest and G2-arrest inhibition, since adavosertib had only moderate effects on the radiation-induced G2-arrest of *TP53* wildtype DKMGvIII−/+ cells and these cells were also radiosensitized (Fig. 4A, B). That Wee1 targeting is an efficient approach to radio- and chemosensitize EGFRvIII− GBM cells, has already been suggested by Mir et al., who showed that EGFRvIII− GBM cells can be pushed into mitotic catastrophe by Wee1 inhibition after DNA damaging radio- or chemotherapy. In their in vitro and in vivo studies, the pre-clinical Wee1 inhibitor PD0166285 was used, which, at higher concentrations, also inhibits CHK1 activity. In the in vivo studies from Mir et al*.,* a U251 orthotopic mouse model was sham irradiated or exposed to a single dose of 6 Gy. A strong tumor progression in both irradiated and non-irradiated mock treated mice at 6 weeks after injection of the cells was observed. Similarly, the non-irradiated PD0166285 treated mice demonstrated profound tumor growth after 6 weeks. In contrary, mice irradiated and treated with PD0166285 exhibited significant tumor regression. Tumor burden was markedly reduced in this animal group, resulting in significantly longer survival. These results demonstrated that pharmacological targeting of Wee1 sensitizes EGFRvIII− U251-derived GBM tumors to irradiation in vivo [9].

First promising clinical results of the combination of Wee1 inhibition and irradiation have already been achieved in a phase 1 clinical trial combining adavosertib with radiotherapy and gemcitabine in pancreatic cancer in line with a previous preclinical study [21–23]. Furthermore, Wee1 inhibition can be combined with other targeting agents, such as PARP-1 or CHK1 inhibitors to further increase radiosensitization efficacy [23–25].

## Conclusion

Our data presented here provide evidence that adavosertib is a promising agent for radiosensitizing approaches that could potentially be added to standard of care treatment for EGFRvIII− and EGFRvIII+ GBM after thorough in vivo testing.

## Supplementary Information

**Additional file 1: Fig. S1.** Heterogenous EGFRvIII and WEE1 expression in human GBM samples. **Fig. S2.** Effect of Wee1 inhibtion on cell cycle distribution. **Fig. S3.** Phosphorylation of CDK1 after Wee1 inhibition. **Fig. S4.** Phosphorylation of CDK1 after irradiation. 

## Data Availability

Not applicable.

## Abbreviations

CDC25: Cell division cycle 25A
CDK1: Cyclin dependent kinase 1
CDK2: Cyclin dependent kinase 2
CHK1: Checkpoint kinase 1
CNS: Central nervous system
CT: Chemotherapy
DSB: DNA double-strand breaks
EGFR: Epidermal growth factor receptor
EGFRvIII: Epidermal growth factor receptor variant III
GBM: Glioblastoma
PARP: Poly [ADP-ribose] polymerase
RT: Radiotherapy
SF4Gy: Surviving fraction at 4 Gy
SF6Gy: Surviving fraction at 6 Gy
