# Supplementary material for: G2 checkpoint targeting via Wee1 inhibition radiosensitizes EGFRvIII-positive glioblastoma cells
Source: Radiat Oncol. 2023 Jan 29;18:19. doi: 10.1186/s13014-023-02210-x (PMC9884419; doi:10.1186/s13014-023-02210-x)
Supplement: Supplementary file 1 — Additional file 1: Fig. S1. Heterogenous EGFRvIII and WEE1 expression in human GBM samples. Fig. S2. Effect of Wee1 inhibtion on cell cycle distribution. Fig. S3. Phosphorylation of CDK1 after Wee1 inhibition. Fig. S4. Phosphorylation of CDK1 after irradiation. [file 13014_2023_2210_MOESM1_ESM.docx]

Fig S1:


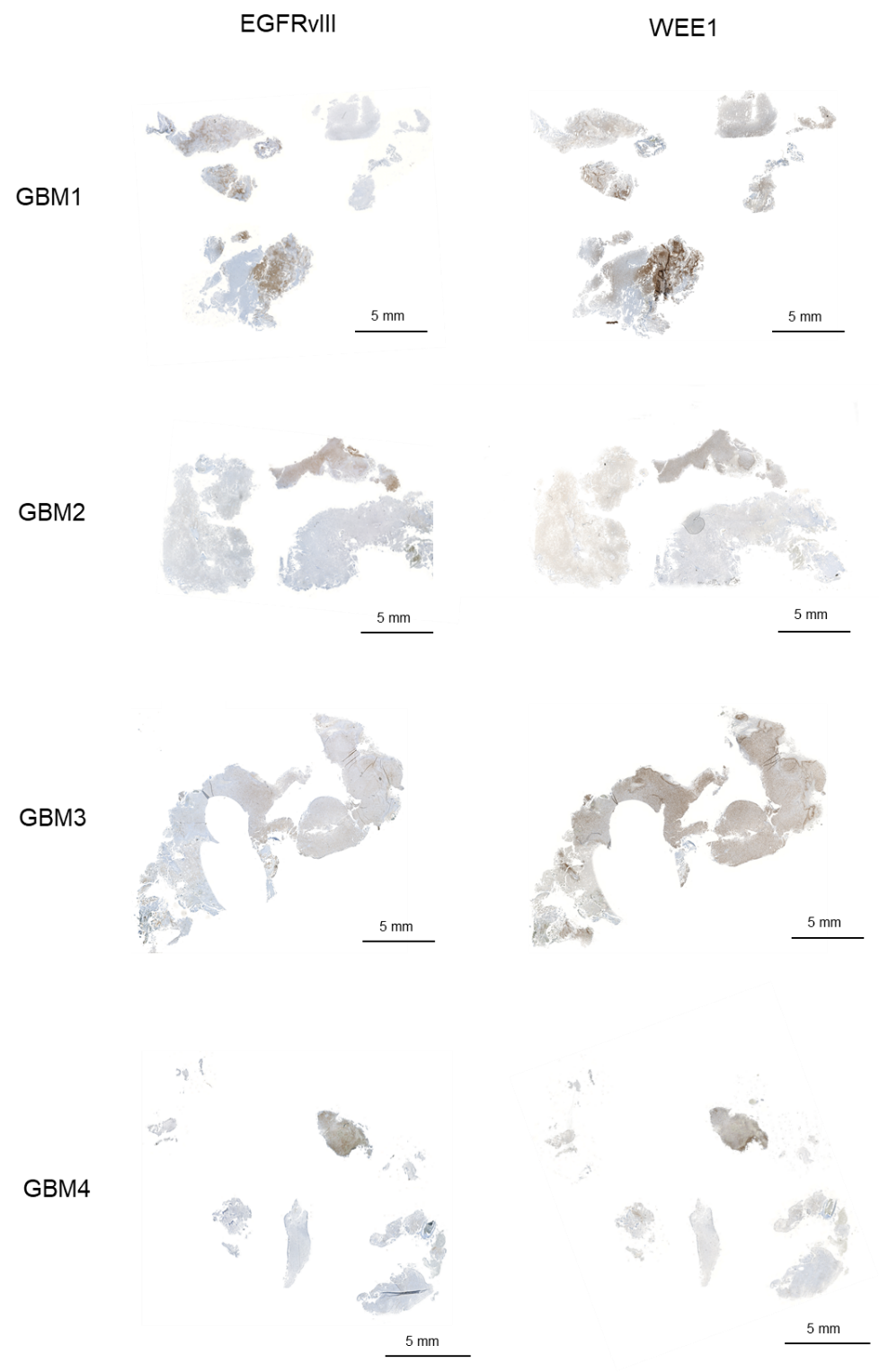


**Fig. S1: Heterogenous EGFRvIII and WEE1 expression in human GBM samples.** Immunohistochemical detection of EGFRvIII in four GBM samples patient samples used for the analysis of WEE1 expression in EGFRvIII- and EGFRvIII+ areas. Scale bars represent 5 mm.

Fig S2:


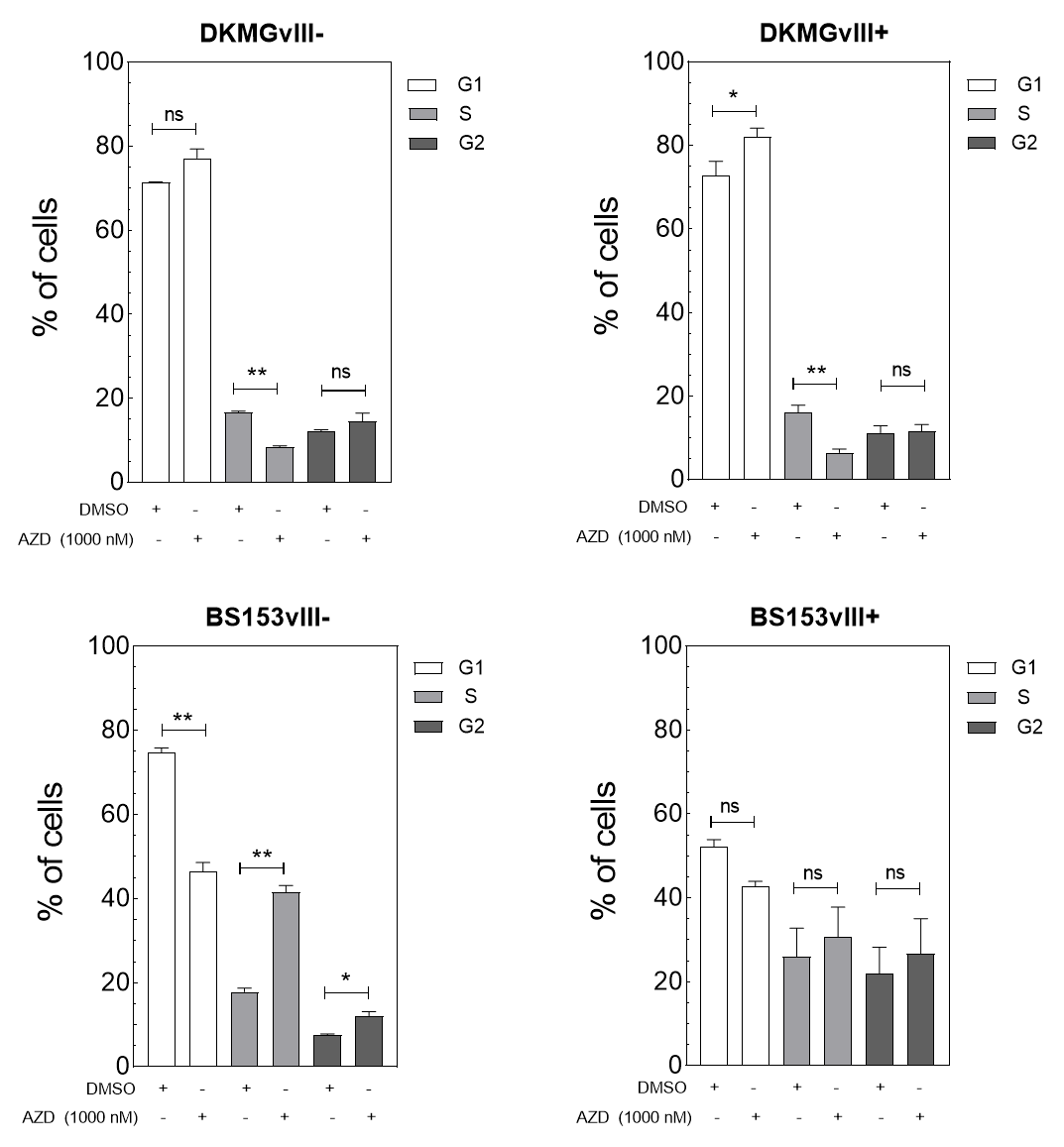


**Fig. S2. Effect of Wee1 inhibtion on cell cycle distribution.** Quantification of cells in G1-, S- and G2-phase after DMSO and adavosertib treatment for 24 h. (n=3; mean with S.E.M; P-values are obtained by using two-tailed Student´s t-test, *p <0.05, **p<0.001; ns: not significant).


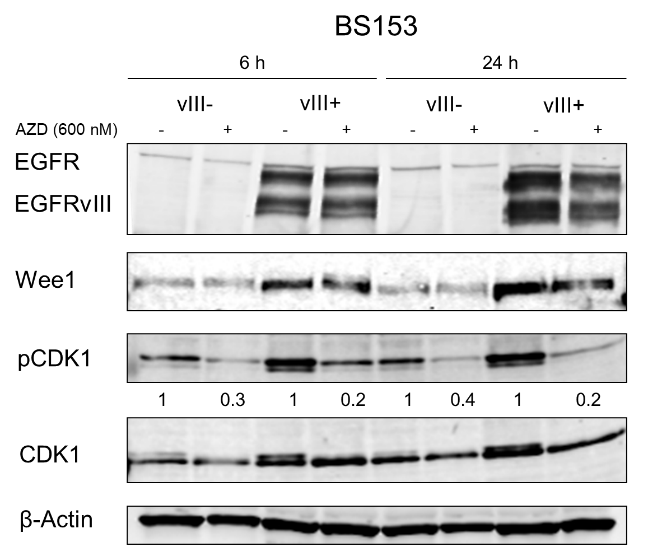
Fig S3:

**Fig. S3. Phosphorylation of CDK1 after Wee1 inhibtion.** BS153vIII-/+ cells were treated with 600 nM adavosertib and harvested after different timepoints as indicated. Expression respectively phosphorylation of CDK1 was assessed by Western blot analysis. β-Actin served as loading control. For quantification of relative CDK phosphorylation after irradiation phosphorylation values were normalized to values of CDK1 expression and to the respective untreated control.

Fig S4:

**Fig. S4. Phosphorylation of CDK1 after irradiation.** DKMGvIII-/+ cells were treated with 6 Gy irradiation and harvested after different timepoints as indicated. Expression respectively phosphorylation of CDK1 was assessed by Western blot analysis. β-Actin served as loading control. For quantification of relative CDK phosphorylation after irradiation phosphorylation values were normalized to values of CDK1 expression and to the respective untreated control.
